# Craniofacial Pain and Disability Inventory and Tampa Scale for Kinesiophobia for Temporomandibular Disorders: A Study of Responsiveness and Minimal Important Change

**DOI:** 10.1111/joor.70140

**Published:** 2026-01-09

**Authors:** Juliana Homem Padilha Spavieri, Mariana Romano de Lira, Aroldo dos Santos Aguiar, Thaís Cristina Chaves

**Affiliations:** ^1^ Graduate Program in Physical Therapy, Department of Physical Therapy Federal University of São Carlos—UFSCar São Carlos Brazil; ^2^ Postgraduate Program in Rehabilitation and Functional Performance, Ribeirão Preto Medical School University of São Paulo Ribeirão Preto Brazil; ^3^ Department of Physical Therapy Federal University of São Carlos São Carlos Brazil

**Keywords:** area under curve, kinesiophobia, patient reported outcome measures, temporomandibular joint disorders

## Abstract

**Background:**

Craniofacial Pain and Disability Inventory (CF‐PDI/Brazil [Br]) assesses Temporomandibular Disorders (TMD)‐related disability, and the Tampa Scale for Kinesiophobia for Temporomandibular Disorders (TSK/TMD‐Br) assesses kinesiophobia in patients with TMD.

**Objective:**

To evaluate the responsiveness and interpretability of CF‐PDI/Br and TSK/TMD‐Br scores in chronic TMD patients.

**Methods:**

A total of 148 participants of both sexes (38, SD = 10.9 years) were included. Participants were reassessed after undergoing a 6‐week rehabilitation treatment. Spearman correlations between change scores of the CF‐PDI/Br and TSK/TMD‐Br were compared with pain intensity and pain self‐efficacy (responsiveness‐construct approach). Accuracy (Area Under the Curve = AUC) was assessed by considering patients who improved or not, using the perceived global effect of improvement scale as an external anchor (responsiveness‐construct approach). Interpretability assessed by minimal important change (MIC) estimate was also calculated using three methods: MIC_ROC_, MIC_mean_ and MIC_predict_.

**Results:**

CF‐PDI and TSK/TMD exhibited suitable responsiveness. More than 75% of the hypothesized correlations between change scores for CF‐PDI and TSK/TMD were confirmed, and both measures showed AUC > 0.70 to distinguish patients who improved from patients who did not improve. We found MIC_predict_ of −5.3 and −4, respectively, for the CF‐PDI and TSK/TMD scores.

**Conclusions:**

CF‐PDI/Br and TSK/TMD‐Br demonstrated appropriate responsiveness and interpretability for patients with chronic TMD. The MIC values presented can be used to evaluate whether improvements in TMD‐related disability and kinesiophobia are clinically meaningful. A change score decrease of at least −5.3 and −4 points in the total CF‐PDI/Br and TSK/TMD‐Br scores, respectively, indicates clinically significant improvement.

## Graphical abstract

Graphical abstract of the manuscript published at the Journal of Oral Rehabilitation, showing the responsiveness and interpretability (Minimal Important Change [MIC]) scores for two questionnaires designed to assess patients with Temporomandibular Disorders: Craniofacial Pain and Disability Inventory (CF‐PDI) and Tampa Scale for Kinesiophobia for Temporomandibular Disorders (TSK‐TMD).

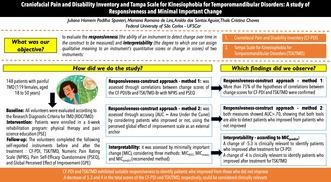

## 1 Background

Temporomandibular disorder (TMD) represents the most common chronic orofacial pain condition, of multifactorial aetiology, more prevalent in women and its peak of prevalence is between 20 and 40 years [1, 2, 3]. Women have a poor TMD recovery prognosis compared to men, with higher rates of transitions from no TMD to any TMD symptoms and a lower rate of recovery [4].

Pain intensity and disability are two of the major outcomes assessed in TMD patients [5]. Craniofacial Pain and Disability Inventory (CF‐PDI/Br) [6] comprehends the assessment of pain, disability and impact of comorbidities in patients with TMD. In addition, there is evidence that kinesiophobia (irrational fear of movement) is a predictor of disability in some musculoskeletal conditions [7, 8] and multiple diagnoses of TMD are associated with higher kinesiophobia scores [9]. The fear‐avoidance model is commonly used to explain how psychological factors affect the experience of pain and the development of chronic pain and disability [10]. It is theorised that negative beliefs about pain lead to catastrophizing thoughts, which in turn may cause disuse, disability and depression, reinforcing the development of persistent pain [11]. Tampa Scale for Kinesiophobia for Temporomandibular Disorders (TSK/TMD‐Br) [12] is commonly used to assess fear of movement in individuals with chronic TMD. A Delphi consensus in TMD reported that TSK/TMD is a recommended tool to be used in TMD [13]. Access to such constructs could bring valuable information to manage patients with chronic musculoskeletal pain like TMD [13].

According to the *Consensus‐based Standards for the selection of health Measurement Instruments* (COSMIN), the quality of a Patient‐Reported Outcome Measure (PROM) should be assessed in three main domains: reliability, validity and responsiveness [14, 15]. COSMIN also suggests the domain of interpretability, that is, the degree to which one can assign qualitative meaning—that is, clinical or commonly understood connotations—to an outcome measure's quantitative scores or change in scores [14, 15]. Responsiveness refers to the ability of an outcome measure to detect change over time in the construct to be measured [14, 16]. It is imperative to assess the responsiveness for evaluative purpose questionnaires, and for questionnaires developed with the aim to capture clinical outcomes [15].

Minimal Important Change (MIC) is considered a measure of interpretability (not a measurement property) [17]. It is an important characteristic for an evaluative purpose instrument [18]. MIC refers to the smallest change in a score that a patient perceives as important [19]. It is useful for clinical purposes and to guide clinical decision‐making since the change in a clinical outcome does not always correspond to a clinically relevant improvement and error‐free measure [20]. Moreover, MIC evaluates the benefit of a certain treatment according to the perception of patient improvement [21].

Both, CF‐PDI/Brazil (Br) and TSK/TMD‐Br, showed acceptable construct validity, structural validity, reliability, internal consistency and measurement error in Brazilian Portuguese, but their responsiveness and interpretability have not been previously reported [9, 12].

Therefore, this study aimed to assess the responsiveness and interpretability of the CF‐PDI/Br and the TSK/TMD‐Br in patients with chronic painful TMD.

## 2 Methods

### 2.1 Participants

A total of 148 patients with painful TMD, of both sexes, 119 females, aged 18 to 50 years (38, SD = 10.9 years), participated in this study. They were consecutively recruited from the Orofacial Pain Outpatient Clinic from the School of Dentistry of Ribeirão Preto, University of São Paulo between May 2019 and May 2021.

This study was submitted to and approved by the ethics committee for research involving human subjects of the Clinics Hospital of the Ribeirão Preto, Medical School of the University of São Paulo (HCFMRP Process No. 3449/2018). All participants gave their informed consent before their inclusion in the study.

The inclusion criteria were as follows: (I) A diagnosis of painful TMD based on the Research Diagnostic Criteria for TMD (RDC/TMD) [22]; (II) a history of orofacial pain at least 6 months before the start of the study; and (III) no cognitive deficits, with a minimal cut‐off score of 22 on the Mini Mental State Examination (MMSE) [23]. The RDC/TMD assessment was conducted by an experienced, trained and calibrated dentist. The RDC/TMD was used in this study because the cross‐cultural adaptation and the measurement properties of the Diagnostic Criteria for Temporomandibular Disorders (DC/TMD) were not available at the time of data collection.

Participants with cognitive deficits, illiteracy, severe depression (medical diagnoses), clinical history of tumours, post‐dental surgery period, infections and with chronic degenerative, inflammatory or neurologic disorders were excluded from this study. Participants with primary headaches were not excluded. Headaches were confirmed according to the International Classification of Headaches [24].

### 2.2 Procedures

All volunteers from both studies were evaluated according to RDC/TMD, conducted by an experienced, trained and calibrated dentist. After being diagnosed by the RDC/TMD, the volunteers answered some questions about the characterisation of the samples and self‐report questionnaires (baseline). After a 6‐week rehabilitation programme including physical therapy and pain science education (PSE) [25], the volunteers answered the self‐report instruments again and the global perceived effect of improvement scale.

The rehabilitation programme consisted of mandibular and neck motor control exercises [25]. The PSE programme consisted of two workshops lasting 45 min, administered at the first two sessions of the protocol. Learning checks that required participants to provide feedback on the material and offer behavioural responses to the material were included. The content covered 17 thematic topics according to the key text, to Explain Pain [26] adapted by the clinical team specifically for TMD. PSE was administered to half of the sample (*n* = 74) [25], which may be appropriate for assessing responsiveness, increasing the chance for including patients who show improvement or do not experience changes in their symptoms.

### 2.3 Instruments Administered in the Study

Craniofacial Pain and Disability Inventory (CF‐PDI/Br) is a self‐administered questionnaire that measures the outcomes of pain and disability related to craniofacial pain. It consists of 21 items, with a total score ranging from 0 to 63 points. Each question is scored on a 4‐point ordinal scale, ranging from 0 to 3. The CF‐PDI has three domains: psychosocial and functioning limitations (items: 2, 4, 6, 8, 11–15 and 21, score range: 0–30), pain domain (items: 1, 3, 5, 7, 10, 19 and 20, score range: 0–21) and co‐morbidities domain (items: 9, 16–18, score range: 0–12). In addition, the total score summing up the scores of the domains can be obtained. Higher scores reflects higher disability levels [9].

Tampa Scale for Kinesiophobia for Temporomandibular Disorders (TSK/TMD‐Br) is a 12‐item self‐report questionnaire that assesses fear of movement. Each item is scored on a Likert‐type scale, ranging from ‘strongly disagree’ (score = 1) to ‘strongly agree’ (score = 4). Features two domains: Activity Avoidance (items: 1, 2, 10, 13, 15, 17 and 18, score range: 7–28) and Somatic Focus (items: 3, 5, 6, 7 and 1, score range: 5–20). Ratings are summed up to yield a total score, ranging from 12 to 48 points. Higher scores reflect a greater fear of movement [12].

### 2.4 Comparator Instruments

The 11‐point Numeric Pain Rating Scale (NPRS) is a simple, easy‐to‐measure scale used to measure pain intensity. Scores could range from 0 to 10 (0 ‘no pain’; 10 ‘worst pain imaginable’) [27]. The instrument demonstrated suitable reliability and construct validity [27].

The Global Perceived Effect (GPE) of improvement scale administered to assess the responsiveness of the CF‐PDI/Br [6] was a single‐item 11‐point scale (ranging from −5 to +5) that compares the patient's current condition with his or her condition at the onset of symptoms. Positive and negative scores are assigned to patients who are better and worse, respectively, and the following question was asked of the patient: ‘Compared to when this episode first started, how would you describe your jaw pain (TMD) these past few days?’ [27]. The instrument demonstrated suitable reliability and construct validity [27].

The pain self‐efficacy questionnaire (PSEQ) [28] assesses self‐efficacy in patients with chronic musculoskeletal pain. It comprises 10 items rated on a scale from 0 to 6, where higher scores (up to 60 points) indicate stronger self‐efficacy beliefs. The instrument demonstrated suitable internal consistency, reliability, construct validity and structural validity [28].

### 2.5 Responsiveness Analysis

Responsiveness is defined as the ability of a PROM to detect change over time in the construct to be measured [15]. In the analysis of the responsiveness‐construct approach, hypotheses of correlations with other outcome measurement instruments raised a priori should be confirmed. COSMIN recommendation [18] suggests that correlations with (changes in) instruments measuring related but dissimilar constructs should range between 0.3 < *r* < 0.5 and correlations with similar constructs *r* > 0.50.

For the responsiveness‐construct approach, we hypothesized the following correlations between the change scores of the CF‐PDI/Br with the NPRS, PSEQ and TSK/TMD scores (Total = 17 hypotheses):

1. Positive correlations (3 < *r* < 0.5) between the change score of the domains (three) of the CF‐PDI/Br and total score versus change in pain intensity scores (NPRS) (4 hypotheses);
2. Positive correlations (3 < *r* < 0.5) between the change score of CF‐PDI/Br (three domains and total score) versus TSK/TMD (two domains and total score) (6 hypotheses);
3. Negative correlations (3 < *r* < 0.5) between the change score of the domains of the CF‐PDI/Br and total score versus change in pain self‐efficacy scores (PSEQ) (4 hypotheses);
4. Positive correlations (*r* > 0.5) between the CF‐PDI/Br total score and its domains' change scores (3 hypotheses).

In addition, for the responsiveness‐construct approach of the TSK/TMD, we hypothesized the following correlations between the change scores of the TSK/TMD with NPRS, PSEQ and CF‐PDI scores (Total = 14 hypotheses):

1. Positive correlations (3 < *r* < 0.5) between the change score of the domains of the TSK/TMD (two domains) and total score vs. change in pain intensity scores (NPRS) (3 hypotheses);
2. Positive correlations (3 < *r* < 0.5) between the change score of the domains of the TSK/TMD and total score versus CF‐PDI (three domains and total score) (6 hypotheses);
3. Negative correlations (3 < *r* < 0.5) between the change score of the domains of the TSK/TMD and total score versus change in pain self‐efficacy scores (PSEQ) (3 hypotheses);
4. Positive correlations (*r* > 0.5) between the TSK/TMD total score and its domain change scores (2 hypotheses).

Responsiveness was deemed suitable if at least 75% of results met predefined hypotheses. In addition, responsiveness was checked with accuracy analysis, using Receiver Operator Characteristic analysis [29, 30]. The Receiver Operating Characteristic (ROC) curve method is based on the ability of a measure to distinguish patients who reported improvement from patients who reported not improved (i.e., stayed the same or worsened) on an external anchor (e.g., GPE as adopted in the current study) [19]. According to the COSMIN manual for systematic reviews, area under the curve (AUC) values over 0.70 indicate sufficient quality of measurement properties [18].

### 2.6 Interpretability

Interpretability is the degree to which one can assign a qualitative meaning to an instrument's quantitative score or change in score [15]. The minimal important change (MIC) was calculated as a parameter of interpretability, which is defined as the smallest change in score in the construct to be measured, which is perceived as important by patients [31]. In the current study, we used an anchor‐based method to assess MIC [32]. We considered the following calculation to determine the change score of the instruments: change score = [posttreatment—baseline assessment] [21].

Patients who reported a GPE score at the end of the treatment ≥ 2 were classified in the group who reported to be improved, and patients with a GPE score < 2 were classified as no change or worsened. In the current study, we adopted three methods to assess MIC: MIC_mean_, MIC_ROC_ and MIC_predict_.

The MIC_mean_ was defined as the mean change score in the subcategory of the subsample of patients who were improved by two units on GPE (anchor score) [19].

The MIC_ROC_ is defined as the value for which the sum of the proportions of misclassifications ([1 − sensitivity] + [1 − specificity]) is smallest [19]. An advantage of this method is that it uses the entire study sample, leading to more reliable estimates than the MIC_mean_. Moreover, it estimates the *threshold* between ‘not changed’ and ‘a little better’ (minimal important improvement).

The MIC_predict_ is defined as the change score associated with a likelihood ratio of 1, which is the change score where the posttest probability of belonging to the improved group (i.e., after knowing the patient's PROM change score) equals the pretest probability of belonging to the improved group (before knowing the patient's PROM change score, the pretest probability is the percentage of improved patients in the sample) [19].

### 2.7 Statistical Analysis

Spearman's rank correlation was used to assess responsiveness—construct approach—by correlating the change scores of the CF‐PDI/Br and TSK/TMD‐Br with the scores of the comparator instruments (NPRS and PSEQ). The magnitude of the correlations was graded according to the pairwise comparisons of the constructs as suggested by Prinsen et al. [18] which was detailed above (Section 2.5).

**FIGURE 1:**
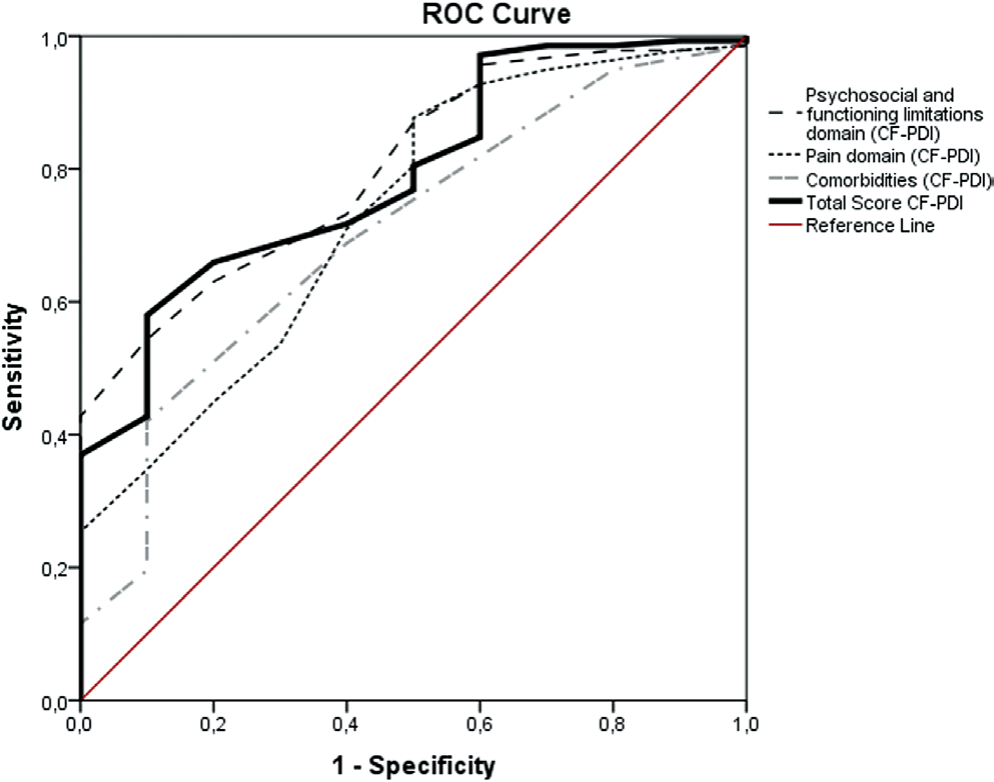
Receiver operating curves (ROC) describing sensitivity and specificity values for accuracy of the scores of the CF‐PDI/Br (Psychosocial and functioning limitations domain score, Pain domain score, Comorbidities domain score and Total score) to detect patients who improved versus patients with no change/get worse.

**FIGURE 2:**
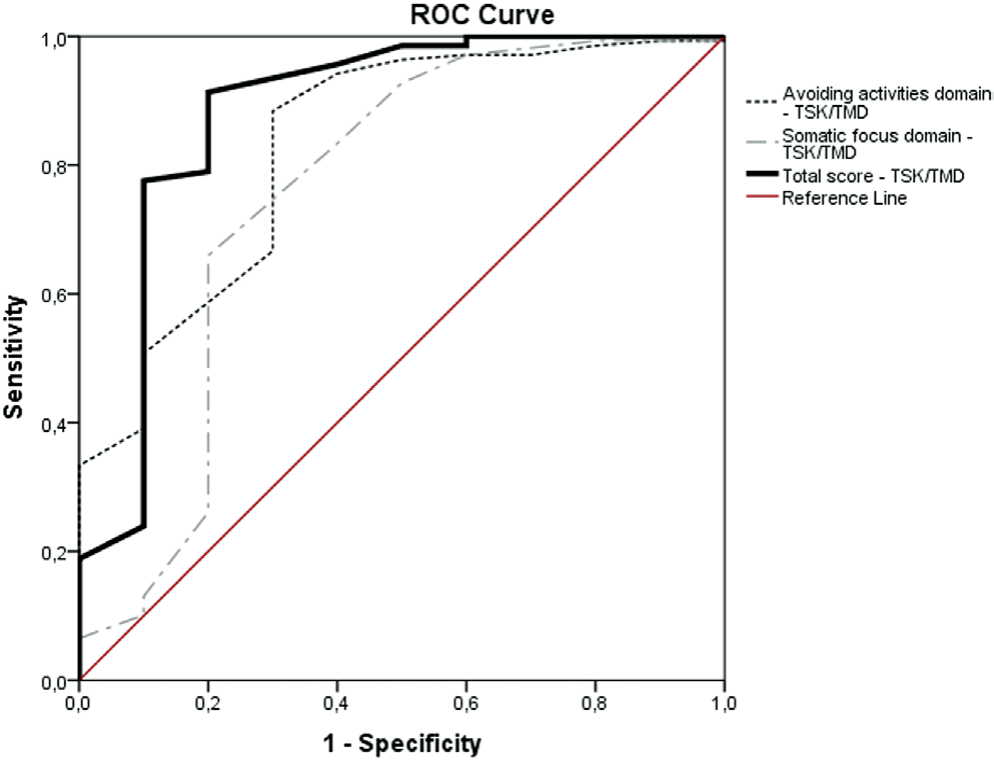
Receiver operating curves (ROC) describing sensitivity and specificity values for accuracy of the scores of the TSK/TMD‐Br (Avoiding activities domain score, Somatic focus domain score and Total score) to detect patients who improved versus patients with no change/get worse.

Additionally, for responsiveness analysis, Receiver Operating Characteristic (ROC) curves were plotted, presenting the Area Under the Curve (AUC) as the probability of correctly discriminating between patients who worsened/remained unchanged or improved (Figures 1 and 2). The AUC, sensitivity and specificity ≥ 0.70 are considered acceptable [33]. A MIC_ROC_ was calculated as the optimal cutoff point in the ROC curve at which the overall classification error rate [(1 − specificity) + (1 − sensitivity)] was the lowest [34]. The MIC_predict_ was calculated considering the recommendation of a previous study [35].

## 3 Results

The sample characteristics of the study can be found in Table 1.

**TABLE 1:** Description (mean and standard deviation: SD) of anthropometric, school level and mean score value of TMD patients recruited for analysis of responsiveness of the CF‐PDI/Br and TSK/TMD‐Br (*n* = 148).

Variables	Mean (SD)
Age (years)	38 (10.9)
Female	119
Weight (kg)	71.3 (13)
Height (m)	1.7 (0.1)
Level of education	
1 = Incomplete/complete basic education	8.8% (*n* = 13)
2 = Incomplete/complete high school	56.1% (*n* = 83)
3 = Incomplete/complete higher education	35.1% (*n* = 52)
MMSE (0–30)	29 (1.1)
Pain intensity baseline (0–10)	6.3 (2.1)
Pain intensity posttreatment (0–10)	2.1 (2)
PSEQ baseline (0–60)	38.9 (13.8)
PSEQ post‐treatment (0–60)	48 (10)
TSK/TMD baseline (12–48)	31.1 (6.4)
TSK/TMD post‐treatment (12–48)	24.3 (6.3)
Psychosocial and functioning limitations domain—CF‐PDI baseline (0–30)	8.9 (5.3)
Psychosocial and functioning limitations domain—CF‐PDI post‐treatment (0–30)	4.8 (3.9)
Pain domain—CF‐PDI baseline (0–21)	8.9 (4)
Pain domain—CF‐PDI post‐treatment (0–21)	4.8 (3.6)
Comorbidities domain—CF‐PDI baseline (0–12)	3.2 (1.8)
Comorbidities domain—CF‐PDI post‐treatment (0–12)	1.9 (1.8)
Total score CF‐PDI/Br baseline (0–63)	21 (9.5)
Total score CF‐PDI/Br post‐treatment (0–63)	11.4 (8.3)

Abbreviations: Br, Brazilian; CF‐PDI, Craniofacial Pain and Disability Inventory; MMSE, Mini Mental State Examination; NPRS, Numerical Pain Rating Scale; PSEQ, Pain Self‐Efficacy Questionnaire; TSK‐TMD, Tampa Scale for Kinesiophobia for Temporomandibular Disorders.

Overall, 138 patients reported improvement greater than two units for the GPE improvement. Table 2 presents the scores pre‐ and post‐treatment and the change scores (post‐treatment score − baseline score) for disability (CF‐PDI) and kinesiophobia (TSK/TMD) in the groups who improved versus did not improve or get worse.

**TABLE 2:** Scores for disability (CF‐PDI) and kinesiophobia (TSK/TMD) were evaluated for subgroups based on whether there was improvement, no improvement/worsening.

Measures	Patients who did not improve/worsening (*n* = 10)			Patients who have improved (*n* = 138)		
	Baseline	Posttreatment	Change score^a^	Baseline	Posttreatment	Change score^a^
Psychosocial and functioning limitations domain	8.4 (4.7)	8.3 (5.3)	−0.1	9 (5.4)	4.5 (3.6)	−4.5
Pain domain	9 (3.8)	8 (5.1)	−1	8.9 (4)	4.5 (3.4)	−4.4
Comorbidities domain	3.8 (1.8)	3.4 (2.4)	−1.4	3.2 (1.8)	1.8 (1.7)	−1.4
Total score CF‐PDI	21.2 (8.4)	19.7 (11.7)	−1.5	21 (9.6)	10.8 (7.7)	−10.2
Activity avoidance domain	16.3 (3.7)	17.5 (3.9)	−1.2	18 (4.2)	13.7 (4)	−4.3
Focus somatic domain	13.5 (4.1)	13.3 (2.8)	−0.2	13.1 (2.6)	10.3 (2.7)	−2.8
Total score TSK/TMD	29.8 (7.7)	30.8 (6.1)	−1	31.1 (6.3)	24 (6.2)	−7.1

^a^ Change score = [post‐treatment score − baseline score].

### 3.1 Responsiveness and Interpretability of the CF‐PDI/Br scores

Our results supported all the hypotheses raised a priori for correlations between CF‐PDI and other instruments except for the pairwise correlations between the Comorbidities domain and pain intensity, pain self‐efficacy and kinesiophobia total score (Table 3). In addition, we confirmed the correlations between the change scores between the domains of the CF‐PDI, with correlations *r* > 0.5. As a result, we confirmed 14 out of 17 hypotheses (82%) for the correlation between change scores of the CF‐PDI and other instruments or within its domains.

**TABLE 3:** Correlations between change scores of the CF‐PDI and TSK (domain and total scores) versus pain intensity change scores and pain self‐efficacy change scores (*n* = 148).

Questionnaires and domains	NPRS	PSEQ	Total score TSK/TMD	Total score CF‐PDI
Psychosocial and functioning limitations domain—CF‐PDI	0.33**	−0.3**	0.45**	0.9**
Pain domain—CF‐PDI	0.47**	−0.34**	0.42**	0.88**
Comorbidities domain—CF‐PDI	0.23**	−0.2**	0.25**	0.64**
Total score CF‐PDI	0.43**	−0.36**	0.48 **	NA
Activity avoidance domain	0.42**	−0.35**	0.91**	0.43**
Focus somatic domain	0.23**	−0.2*	0.81**	0.35**
Total score TSK/TMD	0.43**	−0.34**	NA	0.48 **

*Note:* Change score = [post‐treatment score − baseline score]. Grey cell = unmet criteria for good quality of measurement properties. Black cell = correlation duplicated.

Abbreviations: Br, Brazilian; CF‐PDI, Craniofacial Pain and Disability Inventory; NPRS, Numerical Pain Rating Scale; PSEQ, Pain Self‐Efficacy Questionnaire; TSK/TMD, Tampa Scale for Kinesiophobia for Temporomandibular Disorders.

* *p* < 0.05 Spearman's correlation.

** *p* < 0.01 Spearman's correlation.

The scores (total and domain scores) of the CF‐PDI showed acceptable accuracy (AUC > 0.70) for discriminating who improved versus who did not improve, which is considered sufficient criterion for responsiveness when anchored by the GPE (Table 4). For the total score of CF‐PDI/Br, the MIC_predict_ showed the smallest value (−5.3) (Table 4).

**TABLE 4:** Accuracy and MIC estimates for disability (CF‐PDI) and kinesiophobia (TSK/TMD) considering patients who improved (*n* = 138) versus patients who did not improve (*n* = 10) according to global perceived effect (GPE).

CF‐PDI	AUC (95% CI)	Score	Sensitivity	Specificity	MIC_ROC_	MIC_mean_ (*n* = 26, patients who improved 2 units on GPE)	MIC_predict_ (95% CI)
Psychosocial and functioning limitations domain	0.8 (0.69–0.92)	−1.5	0.70	0.60	−3.5	−3.1	−1.9 (−6.4 to 0.8)
Pain domain	0.73 (0.57–0.89)	−2.5	0.71	0.60	−2.5	−2	−2.7 (−9.9 to 0.9)
Comorbidities domain	0.7 (0.54–0.86)	−1.5	0.42	0.90	−1.5	−1	−1.7 (−3.5 to 0.2)
Total score CF‐PDI	0.8 (0.67–0.92)	−6.5	0.70	0.80	−6.5	−6.1	−5.3 (−8.1 to 1.2)

TSK/TMD	AUC (95% CI)	Score	Sensitivity	Specificity	MIC_ROC_	MIC_mean_	MIC_predict_
Activity avoidance domain	0.83 (0.69–0.97)	−2.5	0.40	0.90	−4.5	−3.3	−1.2 (−4.6 to 0.8)
Focus somatic domain	0.76 (0.55–0.96)	−1.5	0.50	0.80	−2.5	−2.3	−1.6 (−4.8 to 0.4)
Total score TSK/TMD	0.81 (0.64–0.98)	−3.5	0.79	0.90	−6.5	−5.7	−4 (−8.5 to −1.8)

Abbreviations: AUC, area under the curve; Br, Brazilian; CF‐PDI, Craniofacial Pain and Disability Inventory; CI, confidence interval; MIC, minimal important change; SD, standard deviation; TSK/TMD, Tampa Scale for Kinesiophobia for Temporomandibular Disorders.

### 3.2 Responsiveness and Interpretability of the TSK /TMD‐Br Scores

Our results supported all the hypotheses raised a priori for correlations between TSK/TMD and other instruments, except for the pairwise correlations between the Focus Somatic domain and pain intensity and pain self‐efficacy (Table 3). In addition, we confirmed the correlations between the change scores between the domains of the CF‐PDI, with correlations *r* > 0.5. As a result, we confirmed 12 out of 14 hypotheses (86%) for the correlation between change scores of the TSK/TMD and other instruments or within its domains.

The scores (total and domain scores) of the TSK/TMD showed acceptable accuracy (AUC > 0.70) for discriminating who improved versus who did not improve, which is considered a sufficient criterion for responsiveness when anchored by the GPE (Table 4). For the total score of the TSK/TMD, the MIC_predict_ also showed the smallest value (−5.3) (Table 4).

### 3.3 Quality of Measurement Properties

Table 5 is a summary of the quality of measurement properties of the CF‐PDI/Br and TSK/TMD‐Br instruments, including data from the current study and additional data regarding measurement properties from two previous studies [6, 12]. According to the quality criteria for the measurement properties recommended [18], all the measurement properties of both instruments could be classified as sufficient, except for the Smallest Detectable Change (SDC—measurement error) of the focus somatic domain of the TSK/TMD, in which SDC = MIC.

**TABLE 5:** Quality of measurement properties of CF‐PDI/Br and TSK/TMD‐Br.

Measurement property	Rating	Criteria	CF‐PDI/Br	TSK/TMD‐Br
Construct validity (hypothesis testing)	+	The result is in accordance with the hypothesis	+ (hypotheses defined were confirmed in 84% of the correlations investigated)	+ (hypotheses defined were confirmed in 89% of the correlations investigated)
	?	No hypothesis defined (by the review team)		
	−	The result is not in accordance with the hypothesis		
Structural validity	+	CTT: CFA: CFI or TLI or comparable measure > 0.95 OR RMSEA < 0.06 OR SRMR < 0.082	+ (CFI = 0.96, RMSEA = 0.05)	+ (CFI = 0.97, RMSEA = 0.07)
	?	CTT: Not all information for ‘+’ reported IRT/Rasch: Model fit not reported		
	−	Criteria for ‘+’ not met		
Reliability	+	ICC or weighted Kappa ≥ 0.70	+ Psychosocial and functioning limitations domain (ICC = 0.97) Pain domain (ICC = 0.95) Comorbidities (ICC = 0.97)	+ Activity avoidance domain (ICC = 0.93) Focus somatic domain (ICC = 0.95)
	?	ICC or weighted Kappa not reported		
	−	ICC or weighted Kappa < 0.70		
Internal consistency	+	At least low evidence for sufficient structural validity AND Cronbach's alpha(s) ≥ 0.70 for each unidimensional scale or subscale	+ Psychosocial and functioning limitations domain (Cronbach's *α* = 0.86) Pain domain (Cronbach's *α* = 0.80) Comorbidities domain (Cronbach's *α* = 0.77)	+ Activity avoidance domain (Cronbach's *α* = 0.78) Focus somatic (Cronbach's *α* = 0.86)
	?	Criteria for ‘At least low evidence for sufficient structural validity’ not met		
	−	At least low evidence for sufficient structural validity and Cronbach's alpha(s) < 0.70 for each unidimensional scale or subscale		
Measurement error	+	SDC or LoA < MIC	+ SDC/MIC Psychosocial and functioning limitations domain: SDC = 2.8 < MIC_ROC_ = 3.5, MIC_mean_ = 3.1 Pain domain: SDC = 2.4 < MIC_predict_ and MIC_ROC_ = 2.7 and 2.5 Comorbidities domain: SDC = 1.2 < MIC_predict_ and MIC_ROC_ = 1.7 and 1.5 Total score CF‐PDI/Br SDC = 5.1 < MIC_predict_, MIC_ROC_ and MIC_mean_ = 5.3, 6.5, 6.1	+/− SDC/MIC Activity avoidance domain: SDC = 2.8 < MIC_mean_ and MIC_ROC_ = 3.3 and 4.5 Total score TSK/TMD‐Br: SDC = 4.3 ;<; MIC_ROC_ and MIC_mean_ = 6.5 and 5.7
	?	MIC not defined		
	−	SDC or LoA > MIC		Focus somatic: SDC = 2.5 = MIC_ROC_ = 2.5 and > MIC_mean_ = 2.3 and MIC_predict_ = 1.6
Responsiveness	+	The result is in accordance with the hypothesis OR AUC ≥ 0.70	+ (hypotheses defined were confirmed in > 75% of analysis) and AUC ≥ 0.7	+ (hypotheses defined were confirmed in > 75% of analysis) and AUC ≥ 0.7
	?	No hypothesis defined (by the review team)		
	−	The result is not in accordance with the hypothesis7 OR AUC < 0.70		
Criterion validity	+	Correlation with gold standard ≥ 0.70 OR AUC ≥ 0.70	NA	NA
	?	Not all information for ‘+’ reported		
	−	Correlation with gold standard < 0.70 OR AUC < 0.70		

*Note:* Rating: ‘+’ = sufficient, ‘−’ = insufficient, ‘?’ = indeterminate. Grey cell = unmet criteria for good quality of measurement properties.

Abbreviations: AUC, area under the ROC curve; CFA, confirmatory factor analysis; CFI, comparative fit index; CF‐PDI/Br, Craniofacial Pain and Disability Inventory—Brazilian Portuguese Version; CTT, classical test theory; ICC, intraclass correlation coefficient; LoA, limits of agreement; MIC, minimal important change; NA, not applicable; RMSEA, root‐mean‐square error of approximation; SDC, smallest detectable change; SRMR, standardised root mean square residual; TLI, Tucker Lewis index; TSK/TMD‐Br, Tampa Scale for Kinesiophobia for Temporomandibular Disorders—Brazilian Portuguese Version.

## 4 Discussion

This study aimed to examine the responsiveness of the CF‐PDI/Br and TSK/TMD‐Br, two condition‐specific instruments used to assess disability and kinesiophobia in patients with chronic TMD. The findings confirmed the hypotheses of correlations between the change scores for both the CF‐PDI/Br and TSK/TMD‐Br instruments when compared with other instruments. Consequently, the instruments demonstrated an adequate responsiveness–construct approach. Additionally, we found an AUC > 0.70 for both CF‐PDI and TSK/TMD scores in distinguishing between patients who perceived improvement and those who reported no change or worsening using an additional method to assess responsiveness‐construct approach. Regarding interpretability, ROC curve values were plotted to assess the probability of correctly distinguishing patients who improved from those who did not improve. The results indicated that MIC = −5.3 and MIC = −4 for the CF‐PDI and TSK/TMD total scores, respectively, implying that clinical improvements in patient status are only deemed relevant when the decrease in TMD‐related disability and kinesiophobia scores were greater than −5.3 and −4, respectively.

The responsiveness‐construct approach was considered adequate for both instruments, by comparing change scores, since more than 75% of the hypotheses were met as recommended by the COSMIN good quality criteria for measurement properties [18]. Our findings supported the hypotheses raised a priori of moderate correlations between change scores of the CF‐PDI/Br when compared to pain intensity, pain self‐efficacy and kinesiophobia constructs. Additionally, for the TSK/TMD‐Br, we also confirmed the moderate correlations between change scores of the TSK/TMD when compared to pain intensity, pain self‐efficacy and disability as assessed by TMD‐related disability. Such results suggest that changes in pain intensity and pain self‐efficacy are correlated with changes in TMD‐related disability and kinesiophobia, and that changes in TMD‐related disability are correlated with changes in kinesiophobia and vice versa. No prior study investigated the responsiveness‐construct approach, assessing the correlation between the change scores of both measures compared with other constructs. Previous studies supported simple correlations (not correlations between change scores) between pain intensity and kinesiophobia versus TMD‐related disability [36, 37], and no previous study compared the scores of the CF‐PDI with PSEQ scores. For TSK/TMD scores, previous studies also showed moderate correlations (0.33 < *r* < 0.53) between TMD‐kinesiophobia versus pain intensity and TMD‐related disability assessed by CF‐PDI [37] and correlations with pain‐related disability and self‐efficacy [38] in patients with TMD.

An alternative method to evaluate responsiveness is accuracy analysis, using the ROC curve approach [39]. This method was also used to assess the responsiveness‐construct approach, by comparing scores between different subgroups (known groups) [30]. In the current study, we subgrouped participants using an external anchor as a reference for clinically perceived changes from the patient's perspective (e.g., GPE). Both CF‐PDI and TSK/TMD demonstrated acceptable responsiveness‐construct outcomes by comparing the subgroups of participants who improved vs. who did not improve, with AUC > 0.70 observed for both overall and domain‐specific scores of these measures [39], suggesting that the scores of both questionnaires can detect clinically important changes over time in the construct measured. Furthermore, our findings endorse the use of both questionnaires for monitoring outcome changes in clinical trials and clinical practice. Our study is the first to investigate the responsiveness of both CF‐PDI and TSK/TMD.

Interpretability is defined as the degree to which one assigns qualitative meaning to a quantitative score [16]. Interpreting the meaning of questionnaire scores is essential for comprehending treatment effects when using PROMs. SDC and MIC can be used as benchmarks for the interpretability of a PROM score to determine whether the observed change in score is relevant from the patient's perspective. Hence, when the SDC is smaller than the MIC, it is possible to distinguish a clinically important change from measurement error with a large amount of certainty [32].

There are several methods to analyse interpretability; in the current study, we used three methods MIC_mean_, MIC_ROC_ and MIC_predict_ [19]. Instead, MIC_mean_ is the most commonly used to estimate MIC. This method has some important drawbacks, as the MIC_mean_ is calculated for the small subgroup of patients who reported to be ‘a little better,’ which results in imprecise MIC estimates [19]. MIC_ROC_ and MIC_predict_ are generally regarded as more suitable than MIC_mean_, because both MIC_ROC_ and MIC_predict_ use the entire study sample in their calculation, leading to more reliable estimates than the MIC_mean_. In addition, MIC_predict_ offers the additional benefit of correcting for bias when the proportion of improved patients deviates from 50% [19]. The MIC_predict_ observed in the study for TMD‐related disability and kinesiphobia were −5.3 and −4, respectively, indicating that reductions in the score of CF‐PDI and TSK‐TMD exceeding these cut‐offs represent significant clinical changes. Our study is the first to make available the parameters of interpretability of both measures. A previous study investigated another measurement property of the CF‐PDI, recognised as hypothesis testing for construct validity, using a ROC curve analysis [40]. The study demonstrated that a CF‐PDI total score larger than (or equal to) 4 showed acceptable ability to distinguish TMD patients from asymptomatic participants, with a sensitivity and specificity of 0.85 and 0.88, respectively and AUC = 0.94.

The evaluation of interpretability should take into account the SDC values. To ascertain whether a chance score at the individual patient level holds clinical significance rather than being merely measurement error, the SDC score must not exceed the MIC score [32]. In the current study, the interpretability of both measures was considered acceptable since SDC<MIC, based on the results of SDC obtained from previous publications [6, 12]. Such a result indicates that CF‐PDI and TSK/TMD score reductions surpassing −5.3 and −4 could not be attributed to error and that it is possible to distinguish clinically important change from measurement error with a large amount of certainty. It is important to notice that the SDC=4.3 for TSK/TMD total score, which is greater than the MIC_predict_ showed in the current study. However, we recommend the MIC_predict_=4 even though it exceeded the error estimate by 0.3, which can be considered too small. The exception was for the somatic focus domain score of TSK/TMD, as we found an SDC of 2.5, which was larger than the MIC_mean_ and MIC_predict_. However, it could be considered a minor limitation given that, in the context of clinical trials, the use of domain scores of PROMs is relatively uncommon. Hence, our findings do not support the use of the score of the somatic focus domain score of TSK‐TMD isolated as a parameter of interpretability.

Finally, the sensitivity and specificity of the scores of both CF‐PDI/Br and TSK/TMD‐Br showed acceptable estimates (> 0.70) [33] only for the total scores of the instruments (−6.5 and −3.5). These values indicate that decreases of 6.5 and 3.5 in the scores of the CF‐PDI/Br and TSK/TMD‐Br, respectively, are suitable to distinguish people who improved from those who did not improve. However, the COSMIN suggests that MIC_predict_ is the appropriate measure to assess the MIC. Hence, we suggest that the scores obtained through this method should be followed when considering the MIC (−5.3 and −4).

Following a comprehensive global analysis of the measurement properties of the CF‐PDI/Br and TSK/TMD‐Br, it has been determined that both instruments fully meet the established quality criteria. It is recommended that both CF‐PDI/Br and TSK/TMD‐Br scores be used to assess changes in outcomes for patients with chronic TMD.

This study has certain limitations. Firstly, responsiveness was evaluated using secondary data from a clinical trial, resulting in only 10 participants who did not report improvement after treatment, using GPE scores as the reference. This small sample size of patients who reported no improvement or worsening may affect the results, as the ROC curve analysis considers the cut‐off score by subgrouping the patients based on improvement and lack of improvement (sensitivity and specificity analysis). Secondly, we reported the MIC for improvement only and not for deterioration. Previous studies suggest that the MIC for improvement may differ from the MIC for deterioration [41]. Thirdly, most of the patients recruited in the study were women. We suggest that future studies should aim for a balance between the sexes when recruiting. Nevertheless, literature recognises that TMD is more common in women than men, with a female‐to‐male ratio of 4:1 [42].

## 5 Conclusions

CF‐PDI/Br and TSK/TMD‐Br demonstrated both appropriate responsiveness, according to two approaches and interpretability for patients with chronic TMD. The MIC values presented in this study can be used to evaluate whether improvements in TMD‐related disability and kinesiophobia are clinically meaningful in patients with chronic TMD. A change score showing a decrease of at least −5.3 and −4 points in the total CF‐PDI/Br and TSK/TMD‐Br scores, respectively, indicate clinical improvement from the patient's perspective, which is unlikely to be due to measurement error.

## Author Contributions

This study was designed by Thaís Cristina Chaves. The experiments were performed by Aroldo dos Santos Aguiar and Mariana Romano de Lira. The data were analysed by Thaís Cristina Chaves. Mariana Romano de Lira, Juliana Homem Padilha Spavieri and Thaís Cristina Chaves had a primary role in preparing the manuscript, which was edited by all the authors. All authors have reviewed and approved the final version of the manuscript and agree to be accountable for all aspects of the work.

## Funding

The authors have nothing to report.

## Conflicts of Interest

The authors declare no conflicts of interest.

## Data Availability

The data that support the findings of this study are available from the corresponding author upon reasonable request.
